# Association of Acid-Suppressive Medication and Antimicrobial Use in Infancy with Food Allergy and Anaphylaxis

**DOI:** 10.3390/jcm14113872

**Published:** 2025-05-30

**Authors:** Mohamad R. Chaaban, Julia T. Tanzo, Shvetali Thatte, Matthew Kabalan, David C. Kaelber

**Affiliations:** 1 Head & Neck Institute, Cleveland Clinic, Cleveland, OH 44195, USA; kabalam2@ccf.org; 2 Cleveland Clinic Lerner College of Medicine, Case Western Reserve University, Cleveland, OH 44106, USA; tanzoj@ccf.org; 3 Case Western Reserve University School of Medicine, Cleveland, OH 44106, USA; svt10@case.edu; 4 The Center for Clinical Informatics Research and Education, The Metro Health System, Cleveland, OH 44106, USA; dkaelber@metrohealth.org; 5 The Departments of Internal Medicine, Pediatrics, and Population and Quantitative Health Sciences, Case Western Reserve University, Cleveland, OH 44106, USA

**Keywords:** proton pump inhibitors, H_2_-receptor antagonists, antimicrobials, food allergy, anaphylaxis, atopic dermatitis

## Abstract

**Background/Objectives:** The incidence of food allergies and other allergic diseases is rising. Emerging evidence links both antimicrobials and acid-suppressive therapy with gut dysbiosis, which is implicated in allergy development. We investigated the relationship between the use of acid-suppressive medications or antimicrobials in infancy and the risk of developing childhood allergic diseases. **Methods:** The US network in the TriNetX platform was used to identify patients prescribed proton pump inhibitors (PPIs), histamine-2 receptor antagonists (H_2_RAs), antimicrobials ≥1, or antimicrobials ≥3 times during their first year of life from October 2015 to January 2022. ICD-10 diagnoses were used to assess two-year outcomes of anaphylaxis, food allergy, and atopic dermatitis. A sub-analysis in gastroesophageal reflux (GERD) patients was also performed. **Results:** Risks of anaphylaxis and food allergy increased with the prescription of PPIs (risk ratio [95% CI], 2.49 [1.40–4.41], 5.33 [4.97–5.71]), H_2_RAs (4.48 [3.43–5.86], 4.21 [4.01–4.41]), and antimicrobials ≥1 (2.41 [2.13–2.72], 1.90 [1.86–1.94]), or ≥3 times (3.69 [3.12–4.37], 2.79 [2.70–2.88]). Risk of atopic dermatitis was increased in both H_2_RA (1.41 [1.35–1.48]) and antimicrobial groups (2.25 [2.22–2.28], 3.35 [3.29–3.41]), but not in the PPI group. In the GERD sub-analysis, anaphylaxis risk was not significantly different, food allergy risk was increased in both PPI (2.30 [2.08–2.53]) and H_2_RA groups (1.77 [1.63–1.92]), and atopic dermatitis decreased in the PPI group (0.76 [0.67–0.85]) but slightly increased in the H_2_RA group (1.11 [1.03–1.20]). **Conclusions:** Exposure to acid-suppressive or antimicrobial medications during infancy was associated with increased risk of food allergy and anaphylaxis in early childhood. In infants diagnosed with GERD, exposure to acid-suppressive medications was still associated with increased food allergy risk.

## 1. Introduction

Childhood food allergy is a public health concern and significantly impairs quality of life [1,2]. Recent studies have highlighted a concerning rise in food-related anaphylaxis, with an estimated 177% increase in food-related anaphylaxis cases reported between 2004 and 2016 [3]. Food allergy is part of the progression known as the “atopic march”, a natural history of allergic disease manifestations that begins with allergic sensitization in infancy and often progresses to atopic dermatitis, food allergy, asthma, and allergic rhinitis [4]. Previous research suggests that epigenome–genome–environmental interactions may cause alterations in the immune system that break down tolerance to specific dietary antigens [5]. These interactions include genetic factors, environmental factors, and diet, all of which can modulate epigenetic mechanisms and the overall gut microbiome, resulting in an altered immune response [6]. Considering the human gut microbiota is established in the first 3 years of life, exposures during these years are likely to shape gut microbiota development and potentially induce dysbiosis [7]. 

Among the environmental factors thought to play a role in the development of food allergies is the prescription of medications such as antibiotics and acid-suppressive drugs. There is mounting evidence linking the use of antibiotics to an increased risk of childhood asthma, allergic rhinitis, atopic dermatitis, and celiac disease, among other conditions [8,9,10]. Similarly, acid-suppressive medication use has been linked to an increased risk of developing several childhood allergic diseases [11,12]. Given that acid-suppressive medications are used to treat gastroesophageal reflux disease (GERD) in infants, it is not entirely understood whether the acid-suppressive medications or GERD contribute to this increased risk. 

In this study, we hypothesize that acid-suppressive or antimicrobial medication use during infancy is associated with increased risk of allergies in early childhood. This study has the following objectives: first, to investigate the relationship between the use of acid-suppressive medications or antimicrobials in infancy and the risk of developing childhood allergic diseases, including food allergies and atopic dermatitis; second, to compare the risk of childhood anaphylaxis in those exposed to these medications with those who were not; and third, to assess the risk of childhood allergic disease and anaphylaxis in patients diagnosed with GERD during infancy, comparing those exposed to acid-suppressive medications with those who were not.

## 2. Materials and Methods

### 2.1. Study Design

We performed a retrospective cohort study using the TriNetX US Collaborative Network. TriNetX aggregates, standardizes, and deidentifies electronic health record data, including basic patient demographics (e.g., age and gender), diagnoses (coded using the International Classification of Diseases, Tenth Revision, Clinical Modification, ICD-10-CM codes), and medications (coded using RxNorm with NDF-RT Drug Classes), from over 60 health care organizations across the US. This retrospective study is exempt from informed consent. The data reviewed is a secondary analysis of existing data, does not involve intervention or interaction with human subjects, and is de-identified per the de-identification standard defined in Section §164.514(a) of the HIPAA Privacy Rule. The process by which the data is de-identified is attested to through a formal determination by a qualified expert as defined in Section §164.514(b)(1) of the HIPAA Privacy Rule. This formal determination by a qualified expert was refreshed in December 2020. This study followed the Strengthening the Reporting of Observational Studies in Epidemiology (STROBE) reporting guidelines [13].

### 2.2. Study Participants and Eligibility Criteria

For the primary analysis control group, all patients with a visit during their first year of life between 1 October 2015 and 1 January 2022 and without the prescription of a proton pump inhibitor (PPI), H_2_-receptor antagonist (H_2_RA), or antimicrobial medication during their first two years of life were included. 1 October 2015 was chosen as a start date based on the timeline of when ICD-10-CM codes became incorporated into the US health system. There were four experimental groups which consisted of patients prescribed a PPI (RxNorm code A02BC), H_2_RA (RxNorm code A02BA), antimicrobial (RxNorm code AM000) at least once, and antimicrobial at least three times during their first year of life between 1 October 2015 and 1 January 2022. These groups were mutually exclusive, with patients prescribed an antimicrobial medication during their first two years of life excluded from the acid-suppressive groups and patients prescribed an acid-suppressive medication excluded from the antimicrobial groups.

For the GERD sub-analysis, all patients with one or more ICD-10 encounter diagnoses for GERD (ICD-10 CM code K21) during their first year of life between 1 October 2015 and 1 January 2022 were included and assigned to one of three groups: those prescribed a PPI during their first year of life following their initial GERD ICD-10 encounter diagnosis, those prescribed an H_2_RA during their first year of life following their initial GERD ICD-10 encounter diagnosis, and those without prescription of either acid-suppressive medication from 0–2 years old (control group). Patients prescribed an antimicrobial medication from 0–2 years old were excluded from all groups in this sub-analysis. Baseline characteristics including sex, race, and ethnicity were recorded for each study group.

### 2.3. Study Outcomes

Three allergic outcomes were assessed: anaphylaxis (ICD-10 CM code T78.2), food allergy (ICD-10 CM code Z91.01), and atopic dermatitis (ICD-10 CM code L20). These outcomes were compared between medically treated groups and control groups at two years from the index date. For the primary analysis, the index date for the control group was the date of first visit and the index date for the medically treated groups was the date of first medication prescription. For the GERD sub-analysis, the index date for the control group was the date of the initial GERD ICD-10 encounter diagnosis, while the index date for the medically treated groups was the date of first medication prescription. Patients with an outcome of interest prior to the respective index date were excluded from analysis.

### 2.4. Statistical Analysis

The TriNetX Analytics platform was used to calculate risk ratios (RRs) and associated 95% confidence intervals (CIs). Because confounding factors in the association between acid-suppressive or antimicrobial medications and allergic outcomes have not been identified, groups were compared without using the TriNetX propensity score matching feature. To further account for potential confounding by demographic factors, we conducted a secondary analysis using the TriNetX propensity score matching feature. Cohorts were balanced 1:1 using a greedy nearest-neighbor algorithm with a caliper of 0.1 pooled standard deviations, matching on sex and race/ethnicity (including White, Black or African American, Asian, and Hispanic or Latino classifications). Balance was assessed using standardized differences and visual inspection of propensity score density plots. After matching, allergic outcomes were re-evaluated between exposure and control groups to determine whether associations persisted after demographic adjustment.

## 3. Results

**Figure 1:**
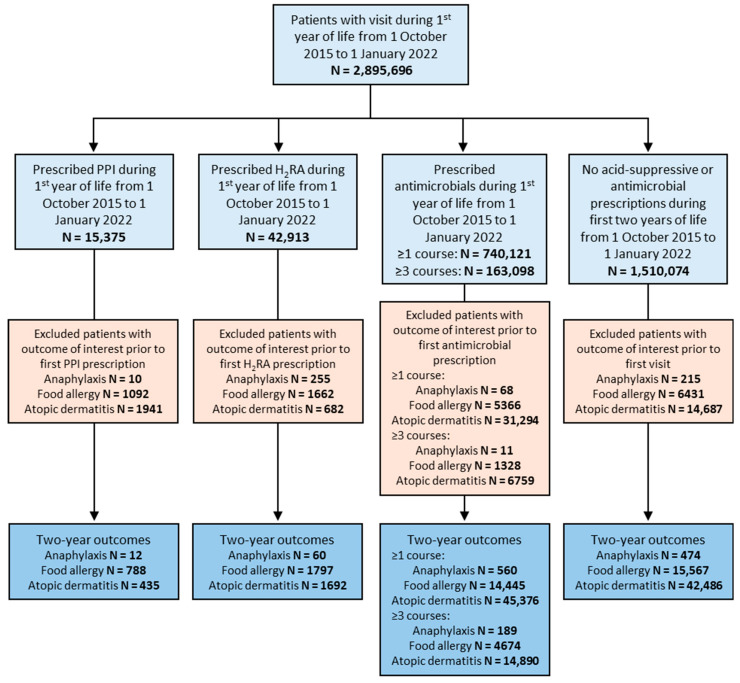
Flow diagram of cohort construction for primary analysis. Acid-suppressive and antimicrobial groups are mutually exclusive. PPI indicates proton pump inhibitor; H2RA indicates histamine-2 receptor antagonist.

Our study included 15,375 patients who were prescribed a PPI, 42,913 who were prescribed an H_2_RA, 740,121 who were prescribed an antimicrobial medication at least once, and 163,098 who were prescribed an antimicrobial medication at least three times during their first year of life (Figure 1). Our control group included 1,510,074 patients who were prescribed neither acid-suppressive medications nor antimicrobials during their first 2 years of life (Figure 1). Sex distribution was similar between groups while racial/ethnic background varied more significantly (Table 1).

**Figure 2:**
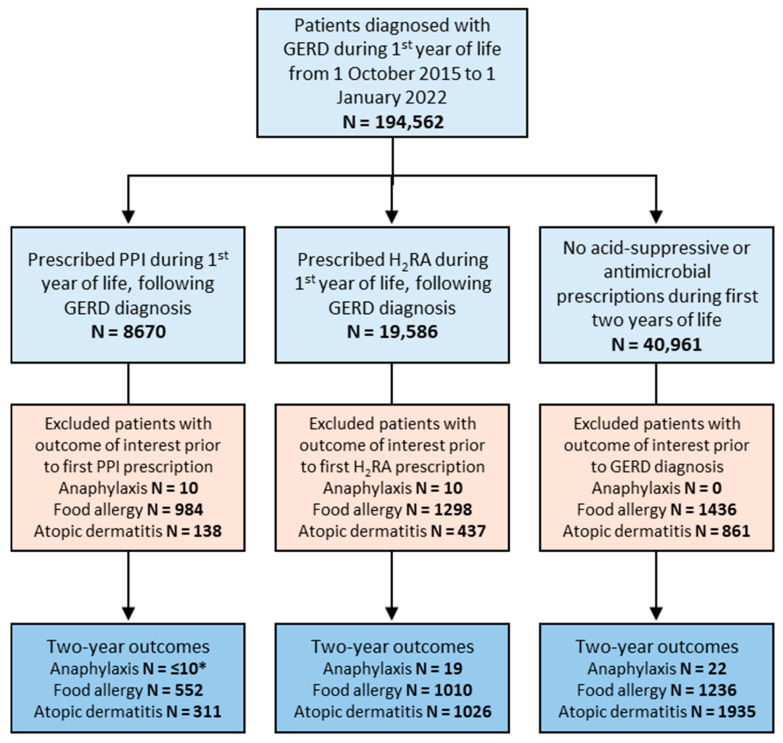
Flow diagram of cohort construction for GERD sub-analysis. Patients prescribed antimicrobials during the first two years of life were excluded from acid-suppressive medication groups. GERD indicates gastroesophageal reflux disease. * To protect patient privacy, TriNetX does not report patient counts <10.

In the subgroup of patients diagnosed with GERD, 8670 patients were prescribed a PPI and 19,586 were prescribed an H_2_RA during their first year of life following their diagnosis of GERD (Figure 2). The control group of GERD patients with no acid-suppressive medication use during their first two years of life included 40,961 patients (Figure 2). Similar to our primary analysis groups, sex distribution was similar between groups while racial/ethnic background varied more significantly (Table 2).

### 3.1. Primary Analysis

**Figure 3:**
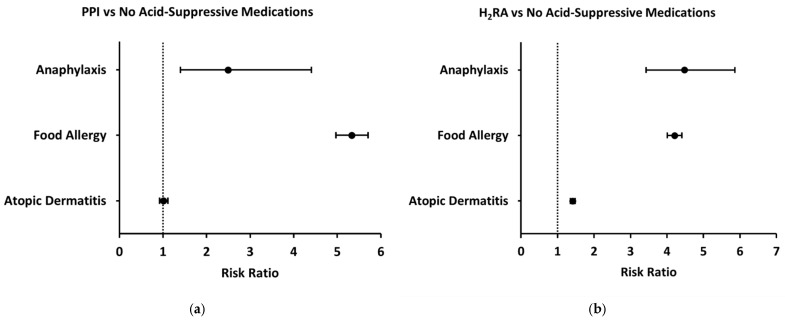
Forest plots demonstrating risk of anaphylaxis, food allergy, and atopic dermatitis in patients prescribed acid-suppressive medications compared to control group (no prescription of acid-suppressive medications). (**a**) Two-year risks of anaphylaxis, food allergy, and atopic dermatitis in patients prescribed PPIs; (**b**) two-year risks of anaphylaxis, food allergy, and atopic dermatitis in patients prescribed H_2_RAs.

Compared to patients without prescription of acid-suppressive or antimicrobial medications during the first two years of life (control group), patients prescribed a PPI during their first year of life had increased two-year risks of both anaphylaxis and food allergy (Unadjusted risk ratio [95% CI], 2.49 [1.40–4.41], 5.33 [4.97–5.71]) whereas two-year risk of atopic dermatitis was not significantly different (1.01 [0.92–1.11]) (Figure 3a). Similarly, patients prescribed an H_2_RA had an increased two-year risk of both anaphylaxis and food allergy compared to the control group (4.48 [3.43–5.86], 4.21 [4.01–4.41]) (Figure 3b). However, these patients also had an increased two-year risk of atopic dermatitis (1.41 [1.35–1.48]) (Figure 3b). In further analyses evaluating specific food allergies and anaphylaxis triggers, both H2RA and PPI exposures were associated with increased odds of reactions to milk, eggs, peanuts, tree nuts and seeds, fruits and vegetables, fish, and other foods. The strongest associations were seen for milk allergy (H2RA OR ≈ 7; PPI OR ≈ 12) and anaphylaxis due to fruits and vegetables (H2RA OR ≈ 13) and fish (H2RA OR ≈ 8). PPI exposure was particularly associated with general food allergy status (OR ≈ 6), milk allergy, and milk-induced anaphylaxis (OR ≈ 5), while H2RA exposure showed broader associations across multiple allergen categories, including egg-induced anaphylaxis (OR ≈ 2–3) and tree nut and seed anaphylaxis (OR ≈ 5). In contrast, associations with seafood and insect allergy were less consistent or not statistically significant (ORs ≈ 1–1). To evaluate whether the observed associations were influenced by demographic factors, we performed a propensity-score-matched analysis controlling for sex and race. After matching, the odds of food allergy among children exposed to PPIs remained significantly elevated—and in fact increased compared to the unadjusted analysis (OR approximately 7). In contrast, the association with anaphylaxis, which was initially significant, was no longer statistically significant after adjustment. The association with atopic dermatitis remained non-significant before and after matching.

**Figure 4:**
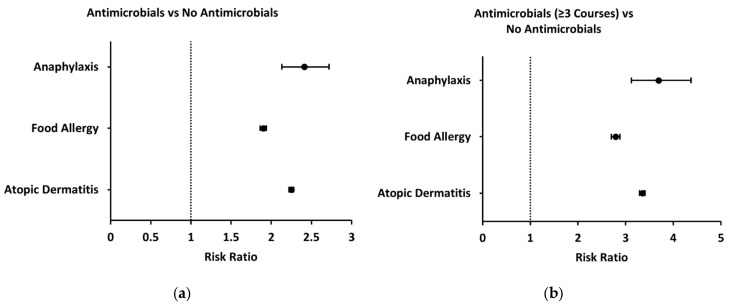
Forest plots demonstrating risk of anaphylaxis, food allergy, and atopic dermatitis in patients prescribed antimicrobial medications compared to control group (no prescription of antimicrobials). (**a**) Two-year risks of anaphylaxis, food allergy, and atopic dermatitis in patients prescribed antimicrobials at least once; (**b**) two-year risks of anaphylaxis, food allergy, and atopic dermatitis in patients prescribed antimicrobials at least three times.

Prescription of antimicrobial medications at least once during infancy was also associated with increased two-year risks of anaphylaxis, food allergy, and atopic dermatitis compared to those without prescription of acid-suppressive or antimicrobial medications during their first two years of life (2.41 [2.13–2.72], 1.90 [1.86–1.94], 2.25 [2.22–2.28]) (Figure 4a). Because antimicrobials are often prescribed multiple times during infancy, we also sought to assess whether a greater number of antimicrobial prescriptions was associated with increased risk of allergic disease. Compared to the control group, those with three or more antimicrobial prescriptions during their first year of life demonstrated an even greater increase in two-year risk of anaphylaxis, food allergy, and atopic dermatitis (3.69 [3.12–4.37], 2.79 [2.70–2.88], 3.35 [3.29–3.41]) (Figure 4b).

### 3.2. GERD Sub-Analysis

**Figure 5:**
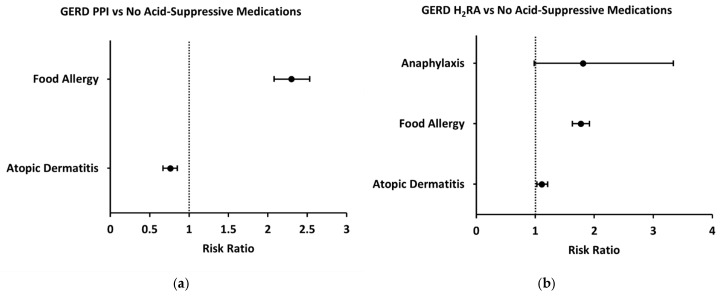
Forest plots demonstrating risk of anaphylaxis, food allergy, and atopic dermatitis in patients prescribed acid-suppressive medications following diagnosis of GERD. (**a**) Two-year risks of food allergy and atopic dermatitis in GERD patients prescribed PPIs; (**b**) two-year risks of anaphylaxis, food allergy, and atopic dermatitis in GERD patients prescribed H_2_RAs.

Because acid-suppressive medications are most commonly prescribed to infants for the treatment of GERD, we performed a subgroup analysis of patients prescribed these medications following their diagnosis of GERD during their first year of life. Compared to GERD patients who were not prescribed any acid-suppressive medications, GERD patients who were prescribed a PPI had an increased two-year risk of food allergy (2.30 [2.08–2.53]) (Figure 5a). Anaphylaxis risk was not assessed in the GERD-PPI subgroup due to a low number of patients with this outcome. With respect to other allergic outcomes, two-year risk of atopic dermatitis was decreased in patients prescribed a PPI following GERD diagnosis (0.76 [0.67–0.85]) (Figure 5a). Similarly, GERD patients prescribed an H_2_RA had an increased two-year risk of food allergy compared to GERD patients with no acid-suppressive prescription (1.77 [1.63–1.92]) (Figure 5b). However, two-year risk of anaphylaxis was not significantly different (1.81 [0.98–3.34]) and risk of atopic dermatitis was slightly increased (1.11 [1.03–1.20]) (Figure 5b).

## 4. Discussion

Our study showed that the use of a PPI or H_2_RA during the first year of life was associated with an increased two-year risk of both anaphylaxis and food allergy, which is consistent with previous studies [11,14]. While patients prescribed PPIs during the first year of life showed no increase in the two-year risk of atopic dermatitis, those on H_2_RAs did experience an increased risk. It has been suggested that acid-suppressive medications may adversely affect gut immune function by altering the gut microbiome, which could influence the development of atopic diseases. For example, certain beneficial gut bacteria produce short-chain fatty acids that enhance the presence of regulatory T (Treg) cells in the intestines, promoting food tolerance. A reduction in these beneficial bacteria may lead to fewer Treg cells and dysregulation in response to food allergens [15]. Because PPIs primarily exert their effects in the gastrointestinal tract, it is likely that they do not increase the risk of atopic dermatitis, as the latter is associated with skin barrier dysfunction and localized immune responses. Comparatively, H2RA use is thought to increase risk of food allergy and anaphylaxis due to a pro-inflammatory effect that increases IgE and IL-5 levels [16,17]. Because histamine also has systemic effects related to skin barrier integrity, H2RAs likely also contribute to an increased risk of atopic dermatitis by disrupting the histamine signaling necessary for expression of proteins involved in skin barrier function [18]. To evaluate whether the observed associations were influenced by baseline demographic factors, we conducted a secondary analysis using propensity score matching for sex and race. After matching, the odds of food allergy remained significantly elevated among children exposed to PPIs (OR approximately 7), indicating that the strong association observed in the primary analysis was not attributable to demographic differences. In contrast, the previously significant association between PPI use and anaphylaxis (unadjusted OR ~ 2) was no longer significant after adjusting for sex and race (OR ~ 1), and the risk of atopic dermatitis remained unchanged and nonsignificant. These findings suggest that some of the initial associations—particularly with anaphylaxis—may have been partially confounded by demographic characteristics. Notably, prior research has documented differences in allergy prevalence and diagnostic patterns across racial and ethnic groups [1,19]. For instance, Black and Hispanic children are more likely to have food sensitization without corresponding clinical diagnoses [19] and may experience underdiagnosis or disparities in access to allergy care [1]. Thus, adjusting for race and sex may help clarify whether observed associations are driven by medication exposure versus broader population-level differences in allergy recognition or reporting.

When sub-analysis was conducted to compare risks of food allergy, atopic dermatitis, and anaphylaxis in GERD patients with and GERD patients without a prescription of acid-suppressive medications (PPI or H_2_RA), we found that GERD patients who had a prescription of a PPI (GERD-PPI) had an increased two-year risk of food allergy and a decreased two-year risk of atopic dermatitis compared to GERD patients with no acid-suppressive prescription. Comparatively, those GERD patients who were prescribed an H_2_RA (GERD-H_2_RA) had an increased two-year risk of food allergy and atopic dermatitis but experienced no change in risk of anaphylaxis compared to those with GERD without any acid-suppressive prescriptions. A prior study demonstrated that prescription of a PPI or H_2_RA were more strongly associated with the development of food allergies than with other manifestations of allergic disease, and in one sense our data corroborate this finding [11]. 

Notably, there was an apparent reduction in the two-year risk of atopic dermatitis in the GERD-PPI group compared to GERD patients who did not receive treatment. These data suggest that another confounding factor in GERD patients may be influencing the association between GERD and the development of allergic diseases beyond just the acid-suppressive medications. It is also important to note that the analysis of anaphylaxis risk in the GERD-PPI subgroup was not performed due to a limited sample size, as TriNetx reported 10 patients or fewer with this outcome, and only 19 patients with anaphylaxis were identified in the GERD-H2RA group.

This study also found an increased two-year risk of anaphylaxis, food allergy, and atopic dermatitis in patients with a prescription of antimicrobials during infancy. The previous literature has investigated the relationship between antibiotic exposure and development of food allergy, with antibiotic prescriptions in children linked to an increased risk of food allergy [20]. One study demonstrated that the duration of antibiotic treatment (≤10 days vs. >10 days) did not seem to correlate to the risk of developing allergic diseases, as both shorter and longer treatment durations were found to have a correlation with these conditions [11]. While our study did not examine differences in the relative risk of developing allergic disease between the various antimicrobials, other literature has suggested that infant exposure to penicillin, macrolides, and cephalosporins is associated with a 30%, 28%, and 19% greater likelihood of experiencing anaphylaxis in the future, respectively [21]. As previously mentioned, the composition of the gut microbiota is strongly associated with allergic manifestations [22], as the commensal bacteria in the gastrointestinal tract promote healthy development of the gut immune system with promotion of food tolerance. Antibiotic exposure disrupts these microbial communities, which in turn affects individuals’ immune response and likely increases their susceptibility for allergic manifestations [23]. Further research is needed to assess the specific microbiome changes which are associated with an increased risk of allergic diseases.

This study’s strengths include its use of data on a very large number of patients from diverse healthcare organizations across various racial, ethnic, and geographic populations, thereby enhancing the generalizability of the findings. Additionally, the utilization of ICD-10 codes represents an improvement over ICD-9 codes utilized in prior similar studies, leading to more accurate and contemporary diagnostic classifications. Despite these strengths, the study has several limitations. One significant limitation is the potential misdiagnosis of food allergies as GERD in infancy. While we attempted to address this by conducting a sub-analysis in GERD patients, this analysis does not fully account for the severity of GERD, which might influence treatment. Infants with more severe GERD symptoms may be more likely to receive acid-suppressive medications, complicating the interpretation of associations between acid-suppressive medication prescriptions and food allergy in GERD patients. However, if the increase in food allergy diagnosis is driven primarily by GERD severity rather than medication exposure, this could offer new insights into the atopic march, suggesting that non-IgE-mediated conditions such as GERD might play a role in early allergic pathways as previously described in the literature [24]. 

## 5. Conclusions

Our study found that the prescription of acid-suppressive medications in infancy is associated with an increased chance of both food allergies and anaphylaxis ICD-10 encounter diagnoses in early childhood. Additionally, antimicrobial prescription was associated with a higher chance of risk of food allergies, anaphylaxis, and atopic dermatitis ICD-10 encounter diagnoses. In a sub-analysis of patients with GERD, the association between acid-suppressive medication prescription and an increased chance of food allergies ICD-10 encounter diagnoses was maintained. While these data are consistent with prior studies, additional research is needed to establish causality and elucidate the biological mechanisms underlying these findings. 

## Figures and Tables

**Table 1:** Baseline patient characteristics.

Characteristic	No. (%)				
	No Medications (N = 1,510,074)	PPI (N = 15,375)	H_2_RA (N = 42,913)	Antimicrobial (N = 740,121)	Antimicrobial ≥3 Times (N = 163,098)
Male	790,734 (52)	8610 (56)	23,672 (55)	393,860 (53)	87,750 (54)
Female	693,150 (46)	6765 (44)	18,964 (44)	341,437 (46)	74,649 (46)
Hispanic or Latino	282,408 (19)	1384 (9)	4924 (12)	165,820 (22)	37,650 (23)
White	732,254 (49)	9994 (65)	26,319 (61)	396,208 (54)	95,809 (59)
Black	244,521 (16)	1845 (12)	5765 (13)	151,400 (20)	32,070 (20)
Asian	64,968 (4)	308 (2)	1385 (3)	27,715 (4)	4545 (3)

**Table 2:** Baseline GERD patient characteristics.

Characteristic	No. (%)		
	No Medications (N = 40,961)	PPI (N = 8670)	H_2_RA (N = 19,586)
Male	21,776 (53)	4794 (55)	10,566 (54)
Female	18,669 (46)	3839 (44)	8850 (45)
Hispanic or Latino	7511 (18)	700 (8)	2279 (12)
White	22,007 (54)	5785 (67)	11,848 (60)
Black	7573 (18)	1004 (12)	2866 (15)
Asian	1122 (3)	195 (2)	573 (3)

## Data Availability

All data is available on the TriNetX platform.

## Abbreviations

GERD	gastroesophageal reflux disease
ICD-10-CM	International Classification of Diseases, Tenth Revision, Clinical Modification
PPI	proton pump inhibitor
H_2_RA	Histamine-2 receptor antagonist
